# Negative reflection and negative surface wave conversion from obliquely incident electromagnetic waves

**DOI:** 10.1038/lsa.2018.8

**Published:** 2018-05-04

**Authors:** Shuo Liu, Tie Jun Cui, Ahsan Noor, Zui Tao, Hao Chi Zhang, Guo Dong Bai, Yan Yang, Xiao Yang Zhou

**Affiliations:** 1 State Key Laboratory of Millimeter Waves, Southeast University, Nanjing 210096, China; 2 Synergetic Innovation Center of Wireless Communication Technology, Southeast University, Nanjing 210096, China; 3 Centre of Intelligent Acoustics and Immersive Communications and School of Marine Science and Technology, Northwestern Polytechnical University, Xian 710072, China; 4 Jiangsu Xuantu Technology Co., Ltd., 12 Mozhou East Road, Nanjing 211111, China

**Keywords:** anisotropic, coding metasurface, negative reflection, negative surface wave, oblique incidence, spatial wave, surface wave

## Abstract

Complete control of spatially propagating waves (PWs) and surface waves (SWs) is an ultimate goal that scientists and engineers seek for, in which negative reflection of PW and negative surface wave are two exotic phenomena. Here, we experimentally demonstrate an anisotropic digital coding metasurface capable of controlling both PWs and SWs with a single coding pattern. On the basis of the digital description of coding metasurfaces, a simple coding method is proposed to allow dual functionalities (either PW or SW manipulations) under two orthogonal polarizations at arbitrarily oblique incidences, thus improving the adaptability of digital coding metasurfaces in more practical circumstances. With elaborately designed ellipse-shaped coding particles, we experimentally demonstrate various functions under oblique incidences, including the negative reflection of PW, negative SW, anomalous reflection and their arbitrary combinations, all having good agreements with theoretical and numerical predictions. We believe that the proposed method may enable the digital coding metasurfaces to have broad applications in radar detections, wireless communications and imaging.

## Introduction

The unprecedented ability of metamaterials to manipulate electromagnetic (EM) waves in desired manners has provided a new route for designing strange devices, which has attracted much interest from both the physics and engineering communities. Many exotic phenomena, such as negative refraction, subwavelength imaging and invisibility cloaking, have been experimentally demonstrated throughout the EM spectrum^1, 2, 3, 4^. Although the emergence of metamaterials and transformation optics offers new approaches for realizing many interesting devices, such as the perfect lens^5^, invisibility cloaks^6, 7, 8, 9^ and antennae^10^, they still rely on the gradient phase accumulation along the beam path through three-dimensional (3D) metamaterials. Inspired by the generalized Snell’s law^11^, ultrathin metamaterials or metasurfaces have been proposed to use discontinuous phase profiles for outgoing EM waves at the interface between two media^11, 12, 13, 14, 15, 16, 17, 18^. The arrays of subwavelength-spaced scatterers, distributed on an ultrathin metasurface with a certain phase gradient, provide an added gradient wave vector, and thus alter the direction of the refracted beam, as determined by the generalized Snell’s law^11, 19, 20^. This phenomenon has been observed experimentally for anomalous reflection and refraction^21, 22, 23^ and has been utilized to generate focused beams^24^ and vortex beams^25, 26^. Some works have recently been reported on the dynamical manipulations of wavefront shaping at microwave frequencies. For example, Chen *et al.*^27^ presents a subwavelength reconfigurable Huygens’ metasurface by loading controllable active elements, demonstrating that complex and multiple focal spots can be dynamically generated in the desired manner. Xu *et al.*^28^ develops a tunable meta-lens by varying external voltages on the incorporated varactor diodes, providing a smart approach to realize the dispersion-corrected and switchable manipulations of EM waves.

Recently, a new concept of a coding metasurface was proposed^29^, from which functional devices are designed in a simple way by encoding a metasurface with appropriate coding sequences^29, 30, 31^. The coding particle is characterized by a certain state from *n*-bit (*n*≥1) digital states instead of the conventional effective medium parameters (permittivity and permeability) with continuous values. The real-time manipulation of wavefronts can also be achieved by loading active components to these digital elements with their digital states being externally controlled by a field-programmable gate array (FPGA), thus realizing digital and programmable metasurfaces^29, 32^. The concept of coding metasurface is not limited only to EM waves but has been extended to the field of acoustic waves. In 2016, Xie *et al.*^33^ presented the first design and experimental demonstration of an acoustic coding metasurface, which could split or focus acoustic waves in a certain bandwidth. On the basis of the similar structure design, the same group developed a multiband asymmetric transmission device for acoustic waves by changing the arrangement of elements ‘0’ and ‘1’^34^. Taking advantage of the broadband operation, a high contrast ratio and the subwavelength thickness, it was remarked that such an acoustic coding metamaterial featuring asymmetric transmission can be used as an acoustic rectifier, and may inspire other ultrathin unidirectional devices in the future.

The digitalization of metamaterials allows us to study them from the perspective of information science, making possible a combination of a coding metamaterial with algorithms in digital signal processing. For example, inspired by the convolution theorem of Fourier transforms, a coding scheme was proposed to steer the radiation beam in an arbitrary direction with negligible distortion by performing the convolution operation on a coding metasurface^35^. On the basis of this coding strategy, a cone-shaped radiation pattern with an arbitrarily designed direction^36^ and a controllable random surface has been presented^37^. It was also reported that the amount of information carried by a coding metasurface can be measured using the Shannon entropy^38^, which reveals the proportional relationship between the entropy of the coding pattern and the entropy of the radiation pattern. However, the isotropic design of the unit-cell geometry brings the same reflection responses under two orthogonal polarizations for each digital state. Therefore, to control the EM waves independently under different polarizations, an anisotropic coding metasurface was proposed in the terahertz domain by designing a dumbbell-shaped coding particle^39^, which shows distinct digital states under two orthogonal polarizations.

Nevertheless, these coding metamaterials and/or metasurfaces, regardless of their structure topology (isotropic or anisotropic) and operating frequency, have been reported only with exotic controls to spatially propagating waves PWs. Surface waves (SWs), as the other common modes of EM waves, have been widely used in the visible light spectrum for sensing applications. Recently, there have been some publications on the conversion from PWs to SWs using graded phase metasurfaces^20, 40^, but they support only one polarization state. In addition, all coding metasurfaces demonstrated in previous literatures, both isotropic and anisotropic, are considered under the normal incidence^29, 30, 31, 32, 35, 36, 37, 38, 39, 41^. In most practical applications, however, oblique incidences are requested. For example, in reflectarray antennas, the feeding source is usually offset from the reflectarray to avoid obstructing the reflected waves in the normal direction, which results in reduced gain and aperture efficiency. The reflection-type coding metasurfaces will also face the same problem when illuminated by a front-fed antenna, limiting its practical applications to ones such as multi-beam radars. Therefore, it is necessary to characterize the performance of a coding metasurface when the EM wave is incident at an arbitrary angle with respect to the normal axis.

Most previously reported metasurfaces for achieving wavefront control share two common points: (1) the functionality is usually designed under the normal incidence, and (2) they belong to isotropic metasurfaces and can only exhibit a single functionality. In this article, we consider a more general scenario, the oblique incidence of an EM wave and propose a compensation method to allow the anisotropic coding metasurface to function the same as one under the normal incidence. More specifically, the coding pattern design at arbitrarily oblique incidences can be simply obtained by adding a compensation coding sequence to the original coding pattern obtained under the normal incidence, which is calculated using the principle of scattering pattern shift^35^. We also propose to control both PW and SW under the arbitrarily oblique incidences of EM waves using 2-bit anisotropic coding metasurfaces in the microwave frequency. It is found that the dual functionalities achieved under the orthogonal polarizations can be used for either PWs or SWs. We emphasize the duplex conversions from PWs to SWs under the oblique incidence scenario, in which the transverse-magnetic (TM) and transverse-electric (TE) polarized PWs are converted to two SWs propagating in different directions. Two interesting physical phenomena are also experimentally demonstrated using the proposed method: (1) negative reflections of PWs, in which the reflected and incident waves are on the same side of the metasurface normal under the oblique incidence of the EM wave; (2) negative SW conversions from the obliquely incident PWs, in which the propagating direction of an SW and incident direction of a PW can be on the same side of the metasurface normal. These two physical phenomena have not been experimentally realized in previous literatures.

## Materials and methods

### Anisotropic coding metasurface with oblique incidence and coding particle design

An anisotropic coding metasurface can be classified as a type of bi-functional coding metasurface that possesses two different functions. The dual functionalities can be realized either through the orthogonality of polarizations or at different frequencies, enabling it to function as a polarization-dependent anisotropic coding metasurface^39^ and frequency-dependent bi-functional coding metasurface^41^, respectively. Prof. Zhou’s group developed a high-efficiency broadband meta-hologram with polarization-controlled dual images using gold cross nanoantennas^42^. The bi-functionality originates essentially from the geometrical anisotropy of the coding particle, which allows each coding particle to have independent coding states under the *x-* and *y-*polarizations. To clearly understand anisotropic coding metasurfaces, we illustrate the working principle in Supplementary Fig. S1 by providing an example of a simple 1-bit anisotropic coding matrix [1/1, 1/0; 0/1, 0/0], in which the digits before and after the slash represent the coding digits under the *x-* and *y*-polarizations, respectively.

**Figure 1:**
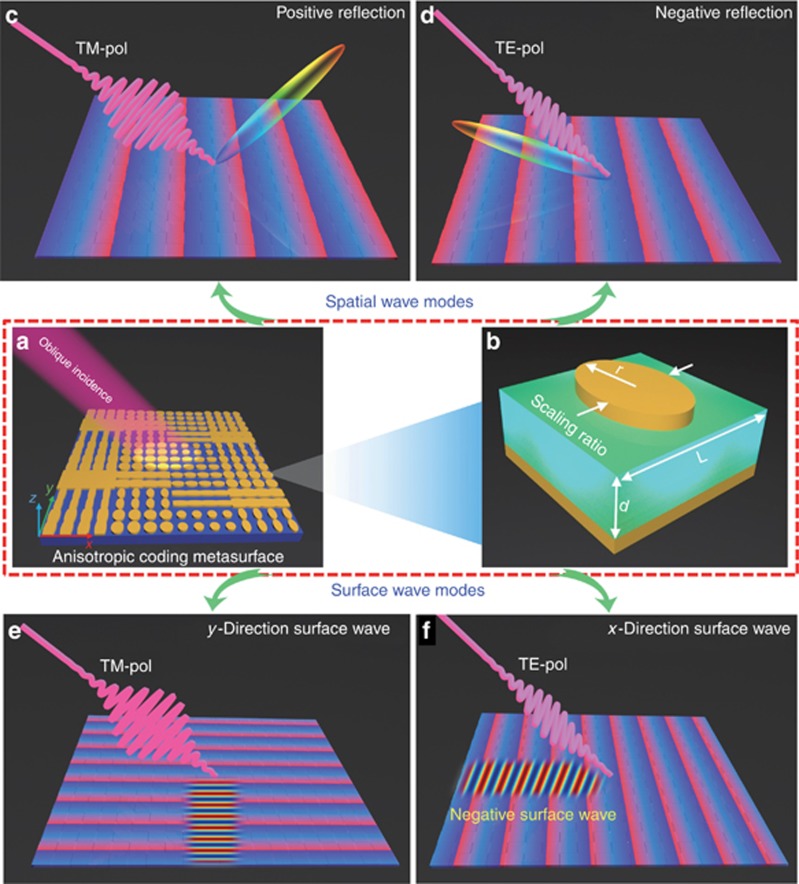
Schematic illustration of the anisotropic coding metasurface in controlling both the PWs and SWs under oblique incidences and the structure design of the anisotropic coding metasurface. (**a**) Schematic of the anisotropic coding metasurface under the oblique illumination. (**b**) Structure design of the anisotropic coding particle. (**c**, **d**) Independent manipulations of spatial waves under the TM and TE polarizations, including negative reflection. (**e**, **f**) Independent manipulations of SWs under the TM and TE polarizations, including the negative SWs.

Figure 1 demonstrates the independent controls of EM waves in both spatial- and surface-wave modes of the anisotropic coding metasurface under oblique incidence. The metasurface is composed of an array of anisotropic coding particles, as shown in Figure 1a and 1b. As each coding particle has dual coding states, the coding pattern of the metasurface is dependent on the polarization of the oblique illumination (see the red light beam). Whether the anisotropic coding metasurface is designed to control PWs or SWs is determined by the coding pattern. Figure 1c and 1d, illustrates the dual manipulations of the obliquely incident wave in the PW mode. For the TM polarization, the oblique incidence is anomalously reflected to the right side (Figure 1c), while it is redirected to the left side for the TE polarization (Figure 1d). It is well acknowledged that the reflected wave should be on the opposite side of the incident wave with respect to the surface normal for conventional specular reflection. However, with proper gradient-coding sequences, both reflected and incident beams can be on the same side of the surface normal (Figure 1d), resulting in an exotic phenomenon of negative reflection^43, 44^. We note that negative reflection has not been demonstrated experimentally previously. Figure 1e and 1f, illustrates the independent manipulations of SWs under the same oblique incidence. Due to the smaller period of the gradient-coding pattern, the obliquely incident waves are converted to SWs propagating along the −*y*-direction (Figure 1e) and –*x*-direction (Figure 1f) for the TM and TE polarizations, respectively. Notably, the SW in Figure 1f propagates in the backward direction with respect to the direction of oblique incidence, resulting in a negative surface wave, which is quite counter-intuitive to what occurs in the conventional spatial-to-surface-wave conversions. In the following sections, we will demonstrate such exotic phenomena with specific examples.

For this purpose, we introduce a new structure as the anisotropic coding particle, which is constructed by printing an ellipse-shaped metallic sheet on the top surface of a commercial FR4 substrate with thickness *d*=2 mm and lattice constant *L*=6 mm, as shown in Figure 1b. The back surface of the substrate is fully covered by a copper layer to guarantee total reflection and zero transmission. The ellipse-shaped metallic structure is obtained by scaling a round disk with radius *r* in the *x*- or *y*-direction with a scaling factor *k*, and then keeping the metallic pattern within the square of the substrate. As only two parameters are required to characterize the coding particle’s geometry, the computational complexity to optimize the parameters of 16 coding particles is effectively reduced. Supplementary Fig. S2 provides locations of the 16 optimized coding particles in the reflection phase diagram, with horizontal and vertical axes representing the reflection phases under the *x*- and *y*-polarizations, respectively. The detailed geometrical parameters for each coding particle are given in Table 1. From Supplementary Fig. S2, it can be seen that the reflection phases under the *x*-polarization (the number before the slash) in each column remain constant and are irrelevant to the digital states under the *y*-polarization (the number after the slash). This is also the case when we look at them from each row, corresponding to independent digital states under the *x*-polarization. To clearly understand the characteristics of the proposed ellipse-shaped anisotropic coding particle, we perform a series of simulations with different geometrical parameters and incident angles, as shown in Supplementary Fig. S3. Such a simple structure with only two tuning parameters can provide sufficient phase coverage and excellent amplitude under two orthogonal polarizations and is stable under oblique incidences. In contrast to the previously proposed coding metasurfaces^29, 30, 31, 32, 33, 34, 35, 36^ that have isotropic designs, the independent coding states under the orthogonal polarizations allow us to encode dual functionalities to a single metasurface.

### Independent controls of spatial waves with out-of-plane reflection and negative reflection

**Figure 2:**
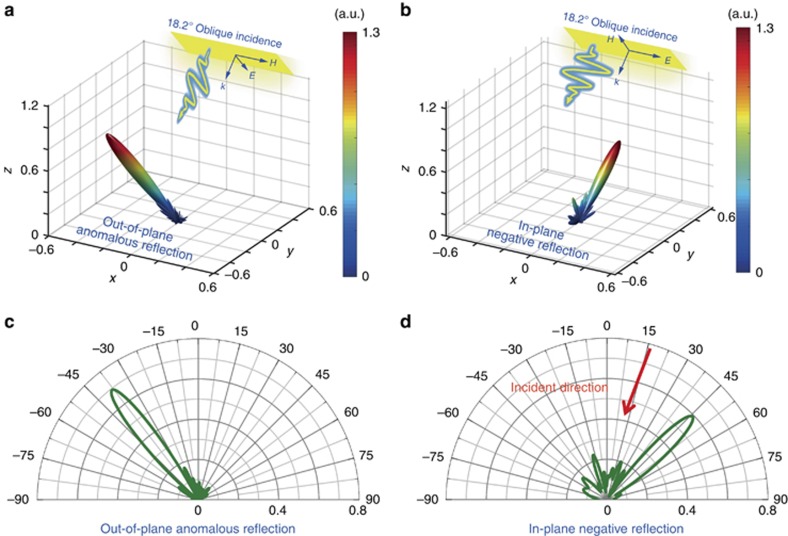
Simulated 3D and 2D far-field scattering patterns of a metasurface encoded with the coding matrix M_1_ at 10 GHz. (**a**, **b**) The 3D scattering patterns under the TM and TE polarizations, respectively. (**c**) The 2D scattering patterns for the TM polarization in the *x–z* plane. (**d**) The 2D scattering patterns for the TE polarization in the *y–z* plane.

We demonstrate the bi-functional performance of an anisotropic coding metasurface in controlling the PWs under oblique incidence. The first anisotropic coding matrix M_1_ consists of 32 × 32 coding particles, which are obtained by combining the sub-coding sequences under the TM and TE polarizations. M_1_ is designed to redirect the TM and TE oblique incidences in anomalous directions. Here, we remark that TE and TM polarizations are used to identify the polarization directions of electric fields for the oblique incidence and the modes of the SWs. The TE and TM polarizations indicate the cases when the electric and magnetic fields are parallel to the metasurface plane, respectively. We introduce a super unit cell (an array of *N × N* identical coding particles) to the coding metasurface to reduce the undesired EM coupling between neighboring coding particles that have different geometries. The advantages of a super unit cell have been clearly elaborated in previous works^29, 30, 31, 32, 35, 36, 37, 38, 39, 41^. Another reason to use a super unit cell is that it allows us to control the radiation direction of the reflected beam by changing the period of the coding sequence. We consider the case when the plane wave is obliquely incident on the metasurfaces at 18.2° with respect to the *z*-axis, as illustrated in Figure 2a and 2b. It is expected that the radiation patterns under both polarizations will be tilted towards the −*y*-direction by *θ*_*r*_=18.2°.

To overcome the rotational effect under the oblique illumination and keep the radiation patterns the same as those obtained under the normal incidence, we propose to compensate the coding sequence under the TM polarization with a gradient-coding sequence [00 01 10 11] along the *y*-direction (the super unit cell is 4 × 4), which corresponds to a single-beam radiation pointing in the reverse direction of the obliquely illuminating plane wave (−*θ*_*r*_=−18.2°). The superposition of the original coding sequence and the compensation sequence can be accomplished by simply calculating the modulus of them by 4. Supplementary Fig. S4a shows the coding pattern M_1_. According to the spatial convolution principle of coding metasurfaces^35^, we know that adding a gradient-coding sequence to a given coding pattern results in a rotation of the original radiation pattern to the designed direction with high fidelity. Because the rotation angle −*θ*_*r*_ is exactly opposite the value of the incident angle, the final radiation pattern will be tilted back to the original direction with negligible distortion. To demonstrate the effect of the compensation method and the polarization-dependent behaviors, we perform numerical simulations for a realistic model using commercial software, CST Microwave Studio. The simulated radiation patterns under TM and TE polarized oblique incidences are shown in Figure 2a and 2b, respectively. For the TM polarization (Figure 2a), the coding pattern is a gradient-coding sequence [00 01 10 11] with a super unit-cell size of 4 × 4, which leads to a single-beam anomalous reflection out of the plane of incidence. Due to the existence of the compensation coding sequence, the beam is not deflected away from the *x–z* plane. For the TE polarization (Figure 2b), the coding pattern becomes the addition (modulus by 4) of two gradient-coding sequences of [11 11 10 10 01 01 00 00…] and [11 11 11 10 10 10 01 01 01 00 00 00…]. According to the spatial convolution principle^35^, the angle of anomalous reflection should exceed 90° for the normal illumination. For the oblique incidence, however, the function to calculate the anomalous scattering angle of the compound coding sequence should be modified to be


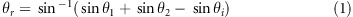


where *θ*_1_ and *θ*_2_ are the anomalous scattering angles of the constitute gradient-coding sequences, and *θ*_*i*_ is the incident angle. The minus sign of *θ*_*i*_ indicates the case when the incident angle is on the same side of the anomalous scattering. From Equation (1), we obtain the negative scattering angle as 46.8°. This is quite a counter-intuitive phenomenon because both the incident and reflected waves are on the same side of the surface normal, leading to the negative reflection.

For clear observations, we plot the two-dimensional (2D) radiation patterns in the *x–z* and *y–z* cutting planes for the TM and TE polarizations in Figure 2c and 2d, respectively. From Figure 2c, we notice an out-of-plane reflection to the angle of −38.5°, which is in excellent agreement with the theoretical prediction of −38.7°. In Figure 2d, the reflection beam points in the negative direction of 46.5°, again being highly consistent with the theoretical value of 46.8°. It is clear that the radiation patterns under both polarizations are located in the expected planes, implying that the proposed compensation technique effectively maintains the radiation patterns unchanged under the oblique incidence. Note that in this case, the incident angle 18.2° is chosen simply because the compensation coding sequence is just the gradient-coding sequence [00 01 10 11] with a super unit cell of 4 × 4. We remark that the oblique angle can be arbitrarily set from 0° to 90°, provided that we find the proper compensation coding sequences. Due to the spatial convolution principle of digital coding^35^, a compensation coding sequence pointing in an arbitrary direction can be readily obtained by calculating the modulus of two gradient-coding sequences with different periods.

To characterize the conversion efficiency of the anisotropic coding metasurface in manipulating the spatial PW, we define the efficiency as the ratio of the intensity of the radiation beam between the sample and a perfectly conducting board having the same dimension. In this case, the efficiency from the oblique incidence to the anomalous reflection and negative reflection can be read from Figure 2c and 2d as 70% and 59%, respectively. However, the conversion efficiency of the anomalous reflection (or negative reflection) can vary depending on different definitions. For example, someone may prefer to use the integrated value of the main beam rather than the largest radiation intensity in the main beam direction. The estimated efficiency would be different as we use different calculation methods and different integration ranges.

### Duplex conversions of spatial waves to surface waves under normal incidence

**Figure 3:**
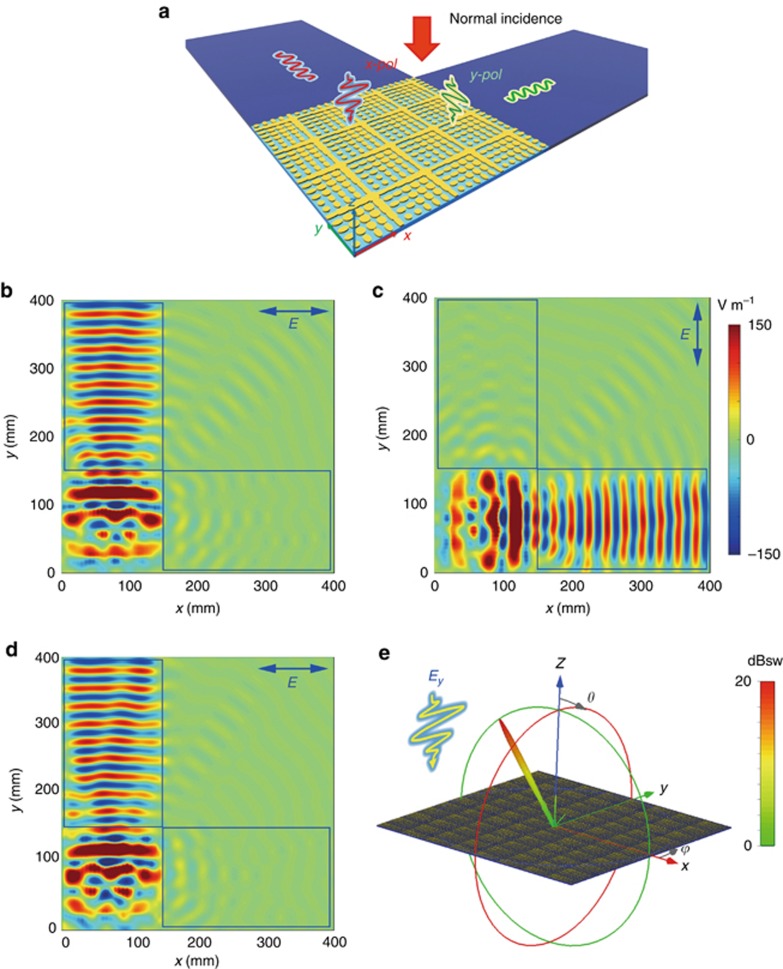
Demonstration for the independent controls of SWs using anisotropic coding metasurfaces under normal incidence at 10 GHz. (**a**) Schematic illustration for the simulation configuration of spatial-to-surface-wave conversion. (**b**, **c**) Electric-field distributions for coding pattern M_2_ under the *x*- and *y*-polarizations, respectively. (**d**) The electric-field distribution (*E*_*x*_) under the *x*-polarized illumination when the coding matrix is M_3_. (**e**) The far-field pattern under the *y*-polarized illumination when the coding matrix is M_3_.

With proper coding sequences, the anisotropic coding metasurface could convert PWs to SWs. Supplementary Fig. S4b shows a coding pattern M_2_ that is a combination of two coding sequences [11 00 01 01 10 11] varying along the *x*- and *y*-directions. Each coding digit is composed of only one coding particle. To demonstrate the PW–SW conversion, we design the simulation model in Figure 3a, where a coding metasurface consisting of 24 × 24 coding particles is placed next to two 4-mm-thick dielectric boards (F4B *ε*_*r*_=2.65, *δ*=0.002), which are used to receive the converted SWs.

We first consider the normal-incidence case, in which a discrete port array is set 80 mm above the coding metasurface to generate the normally incident wave. Figure 3b shows the electric-field (*E*_*x*_) distribution on the *x–y* plane at 10 GHz when the incident wave is polarized along the *x*-direction. It is clearly seen that the PWs is converted to a TE-mode SW propagating along the *y*-direction on the left dielectric board. However, as the incident wave changes to the *y*-polarization, the converted SW changes its propagating direction to the *x*-axis, as illustrated in Figure 3c. The significant difference between SWs propagating in the *x*- and *y*-directions indicates excellent isolation of the two functions under the *x*- and *y*-polarizations. In fact, a gradient-coding sequence can be arbitrarily designed to control the propagating directions of SWs under orthogonal polarizations. We remark that such an anisotropic coding metasurface with independent conversion of PWs to SWs in arbitrary directions can be used as a compound device for surface-beam splitting.

To further demonstrate the excellent isolation of the dual functions of the anisotropic coding metasurface under orthogonal polarizations, we give another example encoded with coding matrix M_3_ (the coding pattern is shown in Supplementary Fig. S4c), in which the coding sequence under the *y*-polarization is [00 00 01 01 10 10 11 11...], whereas the coding sequence under the *x*-polarization remains the same as M_2_. In this case, the normally incident PW is converted to an SW under the *x*-polarization (Figure 3d) but is anomalously deflected to −39° in free space under the *y*-polarization (Figure 3e). Although the coding sequence under the *y-*polarization is different from that of M_2_, this coding metasurface (M_3_) generates nearly the same SW as that in M_2_ when the incident wave is *x*-polarized, as can be observed in Figure 3d. For the detailed 2D radiation pattern, please refer to Supplementary Fig. S5.

### Duplex conversions of spatial waves to surface waves under oblique incidence

**Figure 4:**
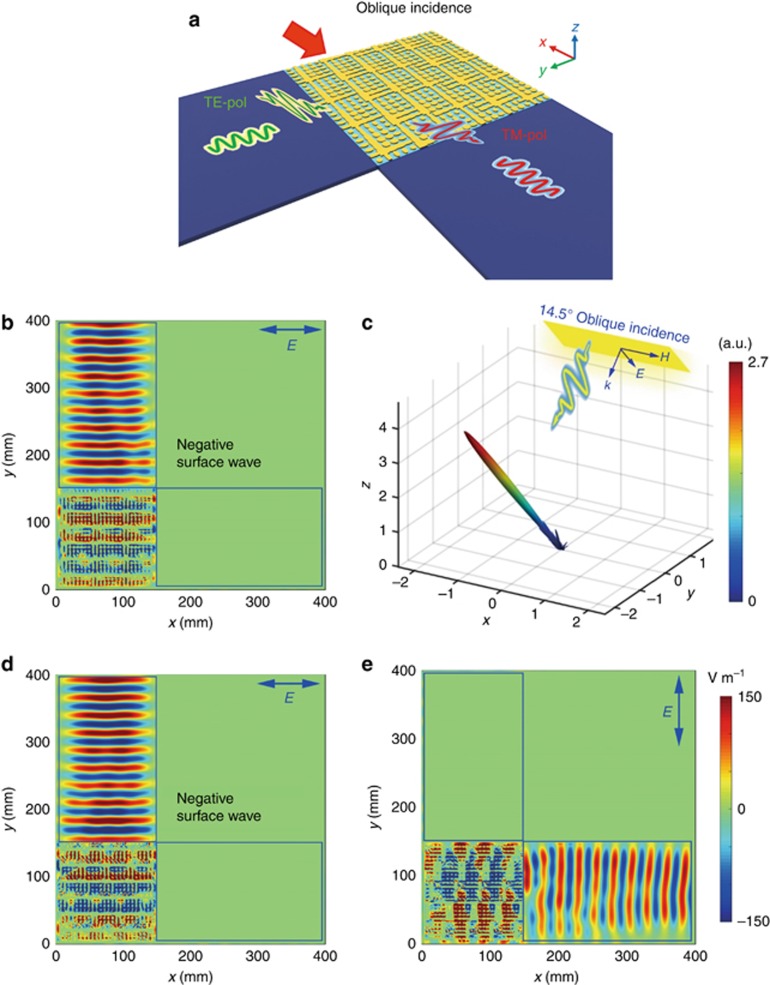
Manipulations of both PWs and SWs using the anisotropic coding metasurfaces with coding matrices M_4_ and M_5_ under the oblique incidence at 10 GHz. (**a**) Schematic illustration of the duplex conversations from spatial waves to SWs under the oblique incidence. (**b**) The electric-field distribution under the TE polarized illumination for the coding pattern M_4_. (**c**) The far-field radiation pattern under the TM-polarized illumination for the coding pattern M_4_. (**d**, **e**) The electric-field distributions for the coding pattern M_5_ under the TE and TM-polarized oblique illuminations, respectively.

Now we consider the general case with an oblique incidence, as illustrated in Figure 4a. To convert the obliquely incident PW to an SW for one polarization, while redirecting it back to free space for the orthogonal polarization, we design an anisotropic coding pattern M_4_, as shown in Supplementary Fig. S4d. Here, the coding sequence under the TM polarization remains the same as M_1_, whereas the coding sequence under the TE polarization is modified to be [00 01 10 11] varying along the *y-*direction. The incident angle is set as 14.5° with respect to the surface normal. Then, the super unit cell size of the compensation coding sequence [00 01 10 11] increases to 5 × 5. We note that only the sub-coding sequence under the TM polarization needs to be compensated. To generate a plane-wave excitation with a flatter wavefront, a 20 × 17 array of discrete ports with a 6 mm spacing is built at 50 mm above the metasurface, as shown in Supplementary Fig. S6a. The simulated radiation pattern under the TM polarization is presented in Figure 4c, which remains the same as that in Figure 2a, with a single beam pointing in the −38.5° direction in the *x–z* plane.

For the TE polarization, the period of the [00 01 10 11] gradient-coding sequence is 24 mm. We give the optimal condition for the PW–SW conversion under the obliquely incident angle *θ* as


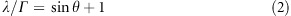


where *Γ* is the period of the gradient-coding sequence. Equation (2) indicates that the incident beam is allowed to have an oblique angle tilted towards the opposite direction of the SW when the period of the gradient-coding sequence is smaller than the wavelength. Inspecting this equation from another perspective, if the oblique incidence tilts towards the propagation direction of the SW, the period of the gradient-coding sequence will increase accordingly. In this case, the obliquely incident PW tilted 14.5° towards the –*y*-direction is converted to an SW propagating along the +*y*-direction, as can be observed from the electric-field (*E*_*x*_ component) distribution on the top surface of the structure in Figure 4b, where we can see that the SW propagates along the +*y*-direction. Such a phenomenon may seem very abnormal if the incident angle further increases because the converted SW unexpectedly propagates in the backward direction, leading to a negative SW.

**Figure 5:**
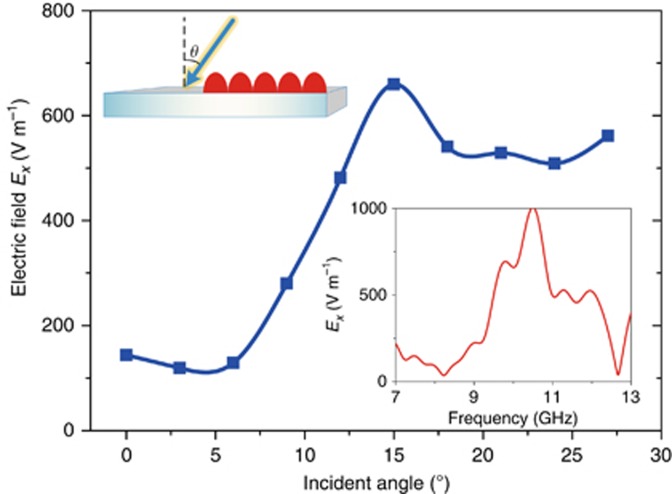
The electric-field intensity measured at the center of the dielectric board at 10 GHz as the incident angle increases from 0° to 27°. The upper-left inset shows the schematic of the simulation configuration. The bottom-right inset provides the intensity of the electric field measured at the center of the dielectric board in broadband under 15° oblique incidence. All plots are obtained with coding pattern M_4_ under the TE polarization.

The performance of PW–SW conversion is further evaluated with different incident angles *θ*. Figure 5 presents the amplitude (*E*_*x*_ component) of an SW probed at the center of the dielectric board at 10 GHz. At the normal incidence (*θ*=0°), the intensity of the SW remains at a low level; it begins to increase as the incident angle exceeds 6° and reaches the maximum at 15°, which matches the theoretical prediction. It is interesting to find that excellent PW–SW conversion is obtained when the momentum of the oblique incidence 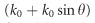 equals the momentum of the graded coding metasurface (*ξ*). It should be noted that, due to the finite-sized discrete port array, the PW–SW simulation results at larger incident angles may not be as accurate as those at smaller incident angles. To guarantee the accuracy of the simulation result, we only provide the amplitude of the SW in the main context from 0° to 27°.

The inset in the bottom-right corner of Figure 5 shows the broadband performance of the converted SW (*E*_*x*_ component) at 15°. Though the coding metasurface is intended to function with 10 GHz waves, the maximum intensity is observed at a slightly larger frequency of 10.5 GHz, which might be attributed to the following reasons. First, the impedance match between the coding metasurface and dielectric board is not flat over the bandwidth of interest. Second, the optimal working frequency of the TE-mode SW is not specially designed to be at 10 GHz with the current thickness of the dielectric board. Third, both inaccurate responses of the realistic structure and a non-ideal plane wave could contribute to the discrepancy. Overall, both the incident angle and frequency attained from the full-wave simulations agree quite well with the theoretical predictions.

We have demonstrated the polarization-dependent feature of an anisotropic coding metasurface in converting PWs to SWs under normal incidence with coding pattern M_2_. In the last example, we evaluate the performance of the proposed compensation technique in such conversions under oblique illumination. The simulation model is illustrated in Supplementary Fig. S6b, in which the TE- and TM-polarized obliquely incident waves are converted to SWs along the +*y* and –*x* directions, respectively. The sub-coding sequence under the TE polarization is identical to that of M_4_, which is [00 01 10 11] varying along the *y*-direction, whereas the sub-coding sequence under the TM polarization is the same as that of M_2_, which is [11 00 01 01 10 11] varying along the *x*-direction. As the incident beam is 14.5° tilted toward the −*y*-direction, a compensation coding sequence [00 01 10 11] (the super unit-cell size is 4 × 4) varying along the *y*-direction is added to the sub-coding sequence [11 00 01 01 10 11]. The anisotropic coding pattern M_5_ is shown in Supplementary Fig. S4e, and the simulated electric-field distributions for the TE and TM polarizations are presented in Figure 4d and 4e, respectively. We observed that the converted SWs propagate in the desired directions, including the negative SW.

To confirm the effect of the compensation coding sequence in offsetting the titled wavefront under the oblique incidence, we purposely simulate the coding pattern M_5_ without adding the compensation coding sequence. Supplementary Fig. S7 shows the simulated electric-field distributions (*E*_*y*_ component) under the TM-polarized oblique incidence. We clearly observe that the SW is shifted in the −*y*-direction, which is the direction that the incidence tilts towards. Comparing Figure 4e with Supplementary Fig. S7, we observed that the wavefront of the SW is finely recovered and propagates in the −*x*-direction; thus, verifying the effectiveness of the proposed compensation coding sequence in overcoming the oblique incidence.

## Results and discussion

A far-field radiation measurement system and a near-field mapping system are employed to experimentally verify the independent manipulations of the anisotropic coding metasurface in both the PW and SW modes. Supplementary Fig. S10a shows the far-field measurement setup in an anechoic chamber. The metasurface sample and a feeding antenna (8–12 GHz) were placed at two ends of a long wooden board, which was mounted on a rotary stage and could automatically rotate 360° to let the receiving antenna (not shown in Supplementary Fig. S10a) record the far-field radiation fields. To provide the required oblique incidence, the metasurface normal was carefully adjusted to the angle of 18.2° with respect to the normal of the receiving antenna. The distance of 1.8 m was maintained to eliminate the phase difference on the metasurface due to the optical path difference between the center and the edge of the metasurface. More details of the experimental configuration can be obtained in Ref. 34.

**Figure 6:**
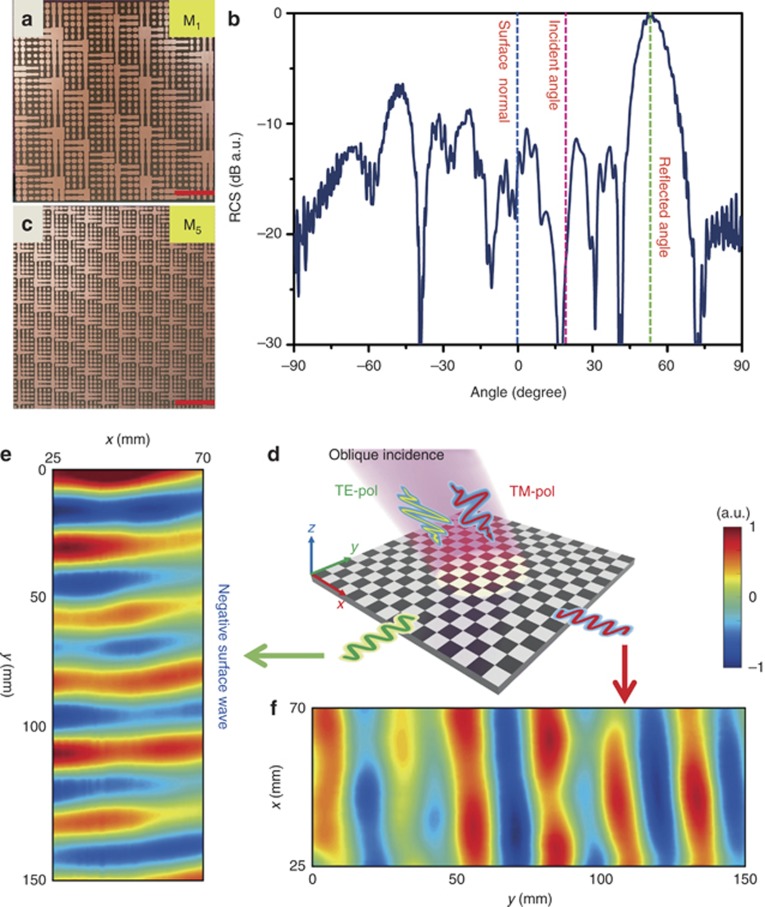
Experimental results for the far-field and near-field measurements. (**a**) The fabricated sample with the coding matrix M_1_. The scale bar corresponds to 60 mm. (**b**) The measured far-field radiation pattern for the sample with coding matrix M_1_. (**c**) The fabricated sample with the coding matrix M_5_. (**d**) The schematic illustration of the duplex conversion from spatial waves to SWs under the oblique incidence in the experiments. (**e**, **f**) The electric-field distributions (*E*_*x*_ component) measured at 10 GHz on the dielectric substrate under the oblique illumination with *x* and *y*-polarizations for coding matrix M_5_. The scanning area is 45 × 150 mm^2^ (*x × y*). The amplitude intensity has been normalized in both plots.

First, we demonstrate the phenomenon of negative reflection experimentally with the sample encoded with coding matrix M_1_, as shown in Figure 6a. The measured radar cross sections (RCSs) of the anisotropic coding metasurface are illustrated in Figure 6b from −90° to +90° under the oblique incidence. An obvious scattering beam is observed at the center angle of 54°, which is obviously the negative reflection. It should be noted that the negative reflection angle obtained in the experiment is slightly larger than that in the numerical simulation, as presented in Supplementary Fig. S8. This is mainly because the metasurface was not placed at the center of rotation, in which configuration the angle between the metasurface and the normal of the feeding antenna is smaller than that when the metasurface is located at the center of rotation. Hence, the measured radiation pattern from −90° to +90° is a portion of the actual radiation pattern, leading to a larger angle of the negative reflection. Overall, we observed very good agreement between the simulated and experimental results.

To measure the polarization-dependent behaviors in converting the PWs to SWs under both normal and oblique illuminations, two samples encoded with coding matrices M_2_ and M_5_ were fabricated using the standard printed circuit board fabrication process on the FR4 substrate, as shown in Figure 6c and Supplementary Fig. S9a, respectively. Both samples include 48 × 48 coding particles and cover an area of 288 × 288 mm^2^. A near-field mapping system was employed to measure the SW propagating on the top of the receiving substrate, as illustrated in Supplementary Fig. S10b. A horn antenna was placed 6 cm above the coding metasurface to provide the normal and oblique illuminations. To keep a clear view of the metasurface in Supplementary Fig. S9b, the distance between the antenna and metasurface was set to be larger than 6 cm. A 4-mm-thick dielectric substrate (F4B *ε*_*r*_=2.65, *δ*=0.002) with a size of 200 × 300 mm^2^ was placed next to the coding metasurface to receive the converted SWs. A microwave probe that is connected to an automatic 2D translation stage is used to scan the electric field (*E*_*x*_) on the dielectric substrate at a height of 1 mm. In the measurement, the probe could automatically scan a rectangular area of 90 × 170 mm^2^ (*x* × *y*).

We first measure the M_2_ sample under normal incidence (Supplementary Fig. S9b). The electric-field (*E*_*x*_ component) distributions measured on the dielectric substrate are plotted in Supplementary Fig. S9c and S9d when the normal incidence is polarized along the *x-* and *y*-directions, respectively. We clearly observe from Supplementary Fig. S9c that the normally incident beam generated from the antenna is converted to a TE-mode SW propagating along the −*y*-direction. In contrast, few SWs are found in the *+x*-direction (Supplementary Fig. S9d). A significant amplitude difference of SWs is observed between the two directions, making the anisotropic coding metasurface a compound device combining both the functions of beam splitting and spatial-to-surface-wave conversion.

Next, we perform the near-field measurements of sample M_5_ to verify the PW-to-SW conversion under the 14.5° oblique incidence (Figure 6d). In this case, Figure 6e and 6f, provides the electric-field distributions (*E*_*x*_) at 10 GHz under the TE- and TM-polarized oblique illuminations, respectively. Comparing Figure 4d and 4e, the duplex conversion physics from PW-to-SW is verified: under the TE polarization, the obliquely incident PW is converted to an SW propagating in the *y*-direction; under the TM polarization, however, the PW is converted to an SW propagating in the *x*-direction. The negative SW is clearly measured. It is noted that such an anisotropic coding metasurface with duplex PW–SW conversions in the microwave frequency could be used as an efficient coupler for the conformal spoof surface plasmon polaritons (SSPPs)^45, 46^, which propagate along a corrugated metal strip with excellent field enhancement and high efficiency, therefore enabling the SSPP structure to receive spatially propagating signals. Most intriguingly, a signal containing two channels of data modulated on orthogonal polarizations can be received by two SSPP structures orientated in different directions using the anisotropic coding metasurface.

## Conclusions

We have experimentally demonstrated a 2-bit anisotropic coding metasurface to manipulate both PWs and SWs under oblique illuminations in the microwave range. More versatile functionalities were realized on a single coding metasurface by encoding it with an anisotropic coding matrix, including anomalous reflections and duplex PW–SW conversions for orthogonal polarizations. We note that a work on anisotropic metasurface was recently reported to exhibit bi-functionality under orthogonal polarizations^47, 48^. However, all simulations and experiments were performed under normal illumination, and only PWs were manipulated in those works. To keep the radiation pattern under oblique incidence the same as that obtained under normal illumination, we proposed an efficient technique to offset the tilted wavefront resulting from the oblique incidence by adding a compensation coding sequence to the original coding pattern. Notably, we have experimentally realized the exotic phenomenon of negative reflection with the reflection angle and incident angle on the same side of the metasurface normal, which may have potential applications in camouflage and illusion optics devices.

Most importantly, we demonstrated experimentally for the first time the duplex conversions from a PW to an SW, which independently convert normally incident waves with *x*- and *y*-polarizations to two SWs propagating in different directions. It is notable that by reducing the period of the gradient-coding sequence below the operating wavelength, the obliquely incident wave can be converted to an SW that propagates in the backward direction, resulting in a negative SW. Such a counter-intuitive phenomenon has never been reported in previous literatures. Experimental results verified the bi-functionalities of the anisotropic coding metasurfaces in controlling PWs and SWs under differently polarized oblique incidences, including the negative reflection of a PW and negative SW conversion. We believe that the proposed anisotropic coding metasurfaces may have broad applications in the fields of radar detections, wireless communications, and microwave circuits.

## Author contributions

SL and TJC contributed equally to this work. SL carried out the analytical modeling, numerical simulations, sample fabrication and measurements. As the principal investigator of the projects, TJC conceived the idea, suggested the designs, planned, coordinated and supervised the work. AN, HCZ, GDB and YY made parts of the numerical simulations, and ZT, and XTZ took part in the sample fabrications and measurements. All authors discussed the theoretical and numerical aspects and interpreted the results. All authors contributed to the preparation and writing of the manuscript.

## Figures and Tables

**Table 1:** Geometrical parameters of the 4 isotropic and 12 anisotropic coding particles

Coding particle	00/00	01/01	10/10	11/11	00/01	00/10	00/11	11/01	11/10	10/01
					01/00	10/00	11/00	01/11	10/11	01/10
*r* (mm)	10	2.72	2.44	1.9	5	5	5	2.9	2.62	2.78
Ratio *k*	1	1	1	1	0.496	0.423	0.296	0.57	0.65	0.85
