# Supplementary material for: Negative reflection and negative surface wave conversion from obliquely incident electromagnetic waves
Source: Light Sci Appl. 2018 May 4;7:18008–. doi: 10.1038/lsa.2018.8 (PMC6060070; doi:10.1038/lsa.2018.8)
Supplement: Supplementary Information [file lsa20188x1.docx]

**Supplementary Information for**

**Negative reflection and negative surface wave conversion from obliquely incident electromagnetic waves**

Shuo Liu^1,2^, Tie Jun Cui^1,2,*^, Ahsan Noor^1,2^, Zui Tao^1,2^, Hao Chi Zhang^1,2^, Guo Dong Bai^1,2^, Yan Yang^1,3^, and Xiao Yang Zhou^1,4^

^1^ State Key Laboratory of Millimeter Waves, Southeast University, Nanjing 210096, China

^2^ Synergetic Innovation Center of Wireless Communication Technology, Southeast University, Nanjing 210096, China

^3^ Centre of Intelligent Acoustics and Immersive Communications and School of Marine Science and Technology, Northwestern Polytechnical University, Xian 710072, China

^4^ Jiangsu Xuantu Technology Co., Ltd., 12 Mozhou East Road, Nanjing 211111, China

^+^ Corresponding author; E-mail: tjcui@seu.edu.cn; Tel: 008683790295; Fax: 008683790295

We first take the 1-bit anisotropic coding as example to illustrate the working mechanism of anisotropic coding metasurface. Supplementary Figure S1 displays the schematic of a 1-bit anisotropic coding metasurface that consists of 8×8 coding particles and is encoded with an anisotropic coding matrix [1/1, 1/0; 0/1, 0/0] (see the middle panel), in which each coding state before and after the slash represents the digital states under horizontal and vertical polarizations, respectively. When it is illuminated by the horizontally polarized wave (see the left panel), it becomes a coding metasurface encoded with [1 0 1 0] coding sequence varying along the vertical direction. In this case, the incident beam splits into two equal beams in the vertical plane. For the orthogonal polarization, interestingly, the equivalent coding sequence is still [1 0 1 0] but varies along the horizontal direction (see the right panel). Different from the horizontal polarization situation, the normally incident beam under the vertical polarization will be deflected in two directions in the horizontal plane. The interference between the digital states under the *x*- and *y*- polarizations is close to zero owing to the excellent geometric anisotropy of the coding particle, which guarantees the independent functions of the coding metasurface under the two polarizations.

The spectral response of the designed ellipse-shaped coding particle is analyzed in Supplementary Figure S3. Figures S3a and b show the phase and amplitude of reflection of the isotropic coding particle when the radius *r* of round metallic patch increases from 1mm to 2.8mm, respectively. It can be clearly observed at the operational frequency 10 GHz that the phase ranges from -105° to 138° with amplitude over 0.5. As the reflection phase ±180° can be readily achieved as round metallic patch touch the edge of the unit cell (i.e., radius>3mm), it is not included in Figure S3. For the anisotropic coding particles, the reflection responses differs when the electric field polarizes along and perpendicular to the compression direction. First, we analyze the reflection responses when the electric field polarizes along the compression direction, as shown in Figures S3c and d. A phase coverage of 198° is obtained with amplitude over 0.88 at 10 GHz as the scaling factor increases from 0.4 to 1.0. The smaller the scaling factor *k* is, the larger the reflection phase will be. The range of phase variation is relatively smaller when the electric field polarizes perpendicular to the compression direction, as is presented in Figures S3e. Because this structure is designed to work under oblique incidence, we analyze its sensitivity under different incident angles, as given in Figure S3g and h. Both the phase and amplitude of reflection experience very little changes as the incident angle increases from 0° to 40°, guaranteeing an accurate performance of the encoded metasurface under oblique incidences.


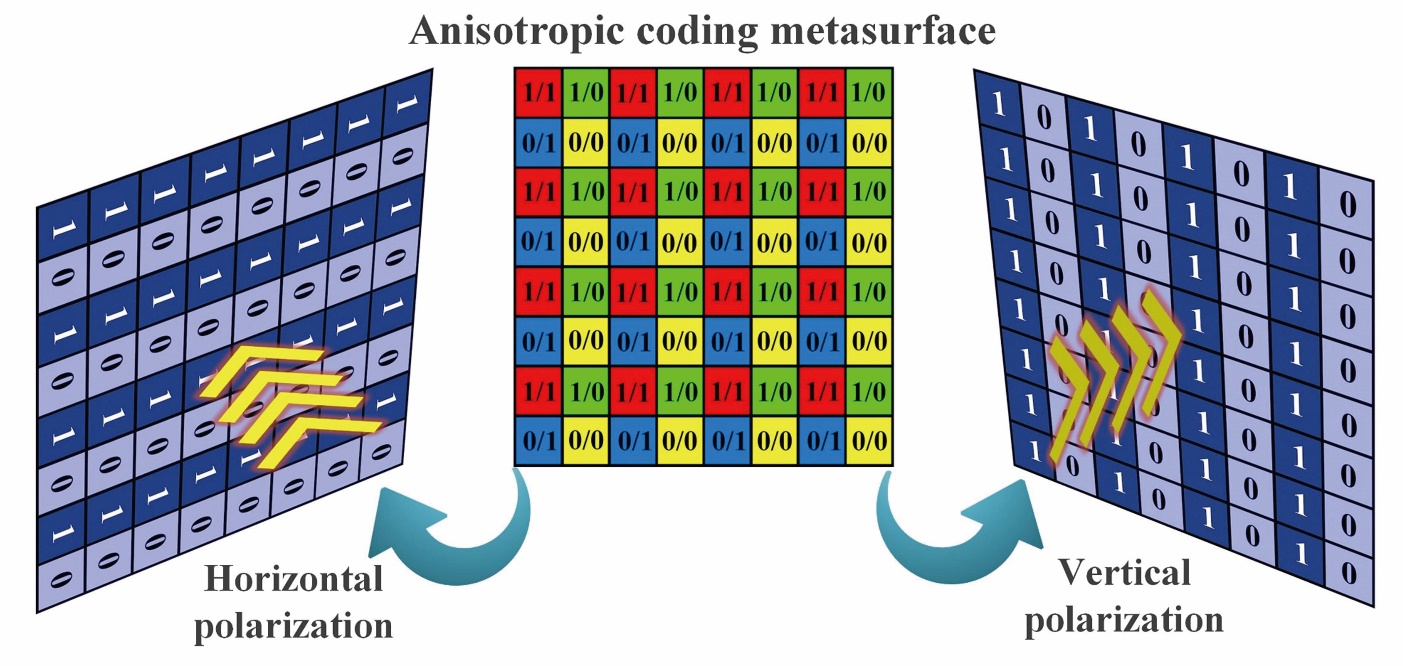


**Supplementary Figure S1 |** **The schematic illustration of a 1-bit anisotropic coding metasurface and its equivalent coding sequences under the horizontal (the left panel) and vertical (the right panel) polarizations, which exhibit different functionalities to the normally illuminating EM wave.**


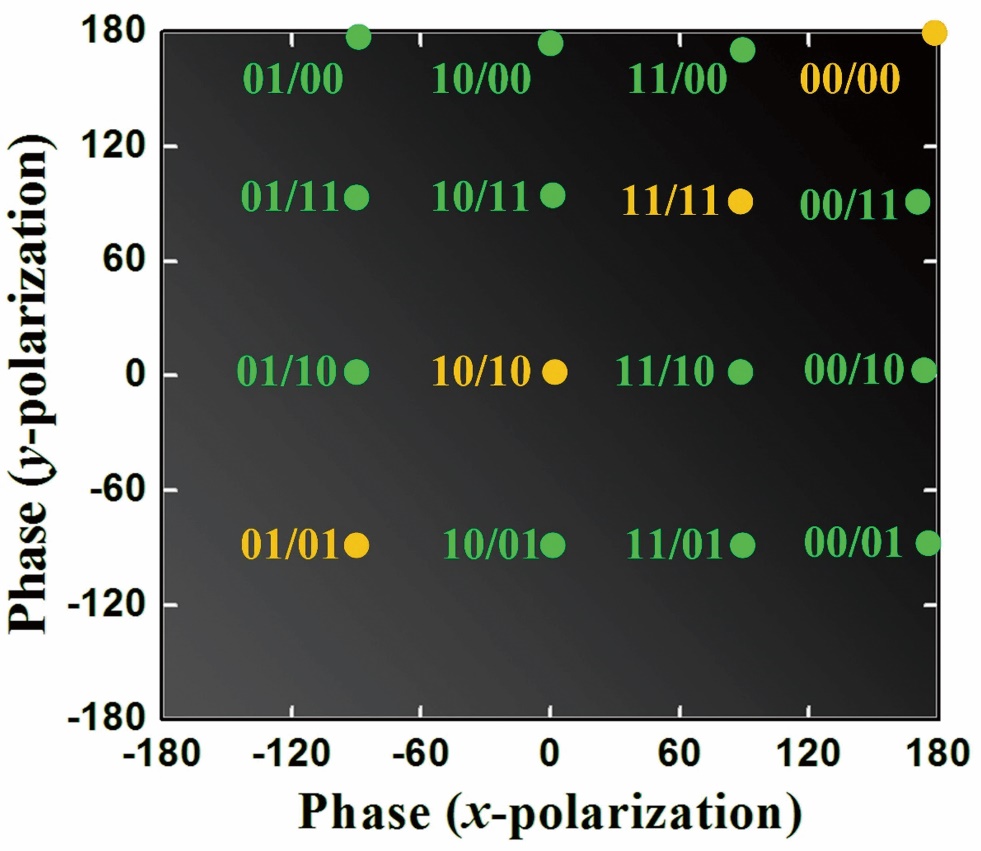


**Supplementary Figure S2 |** **The locations of the 16 coding particles in the reflection phase diagram, with the horizontal and vertical axes representing the *x*- and *y*-polarizations, respectively.**

**
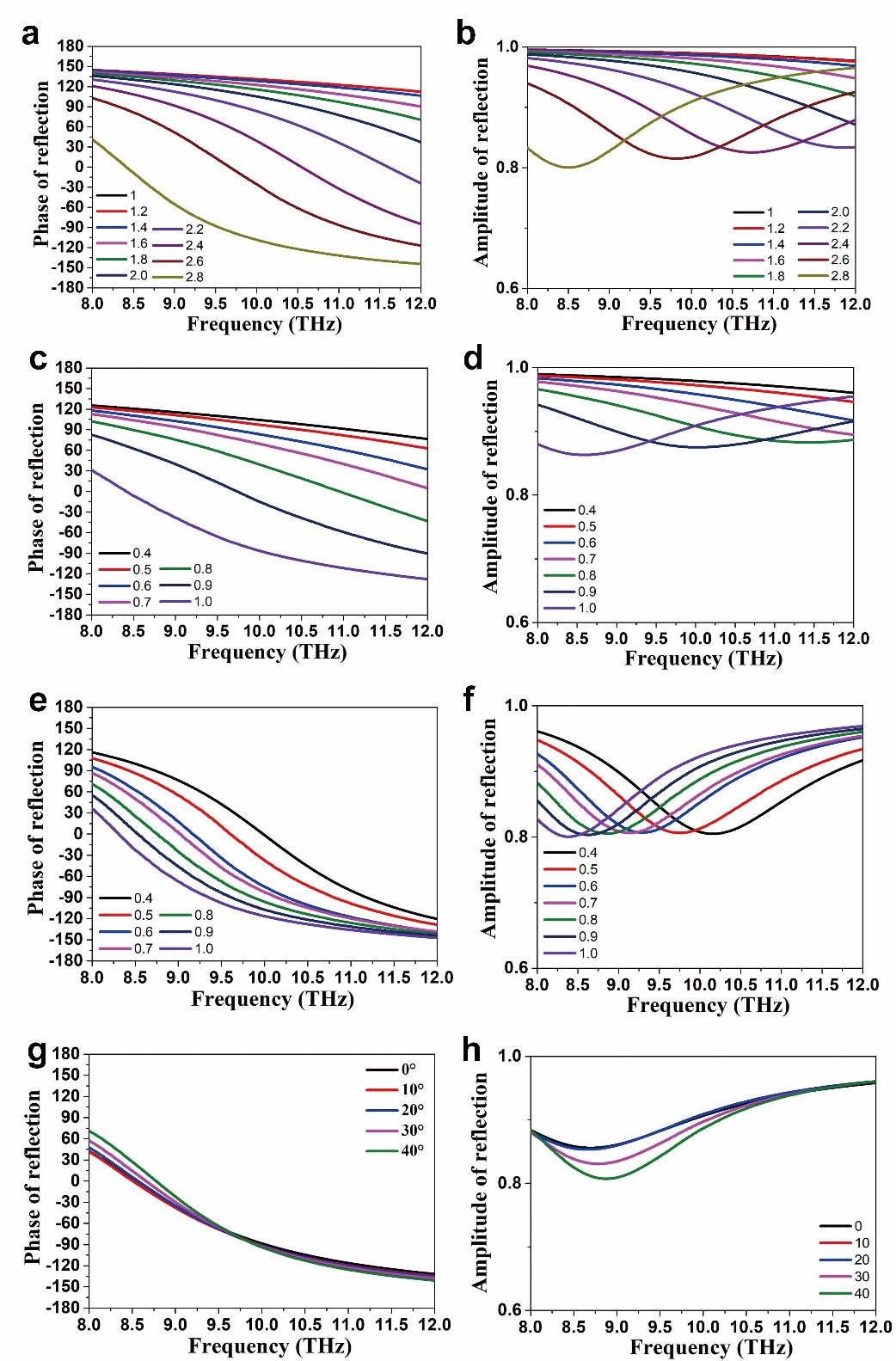
**

**Supplementary Figure S3 | Spectral response analyses of the coding particle with different geometrical parameters.** **(a,b)** Phase and amplitude of reflection as the radius *r* of round metallic patch increases from 1mm to 2.8mm, respectively. Ratio *k* is set to 1. **(c,d)** Phase and amplitude of reflection when the electric field polarizes along the compression direction for ratio *k* ranging from 0.4 to 1.0, respectively. The radius *r* is set to 2.8mm. **(e,f)** Phase and amplitude of reflection as the electric field polarizes perpendicular to the compression direction for ratio *k* ranging from 0.4 to 1.0, respectively. The radius *r* is set to 2.8mm. **(g,h)** Phase and amplitude of reflection of the coding particle when the incident angle increases from 0° to 40°, respectively. Radius *r* and scaling factor *k* is set to 2.72 and 1, respectively, which corresponds to the coding particle 01/01.


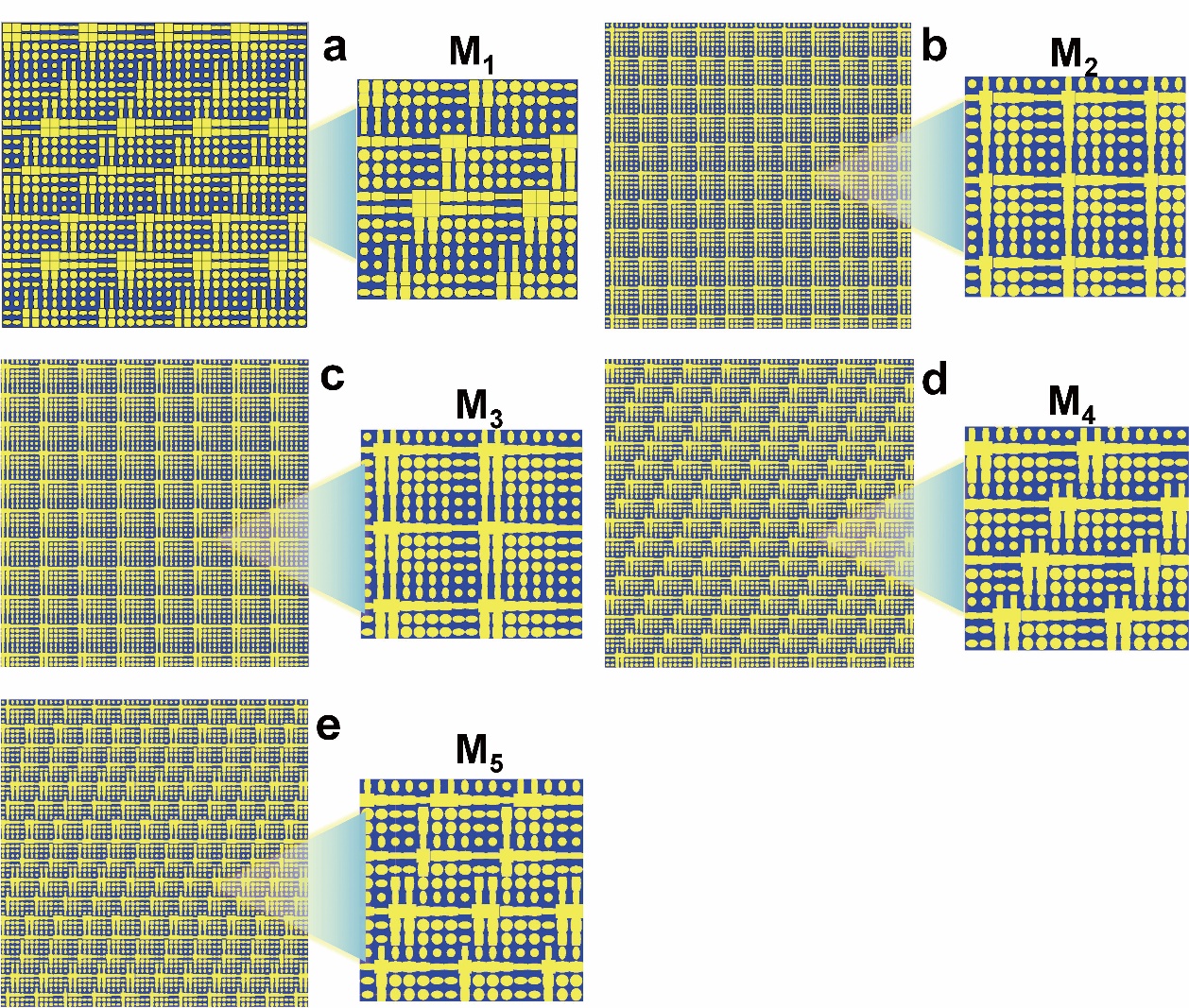


**Supplementary Figure S4 |** **Coding patterns for the anisotropic coding metasurfaces encoded with coding matrices** **(a)** M_1_, **(b)** M_2_, **(c)** M_3_, **(d)** M_4_, and **(e)** M_5_.


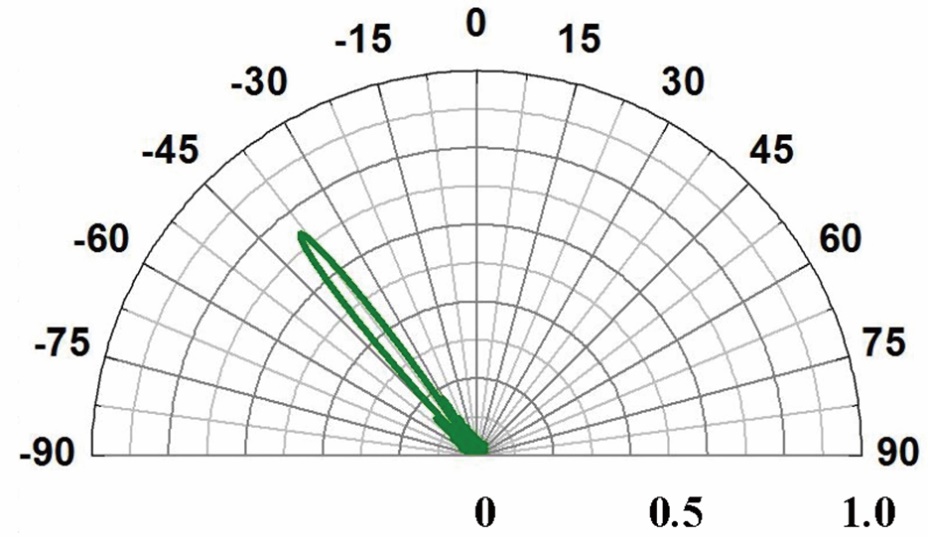


**Supplementary Figure S5 | The 2D scattering pattern (*x-z* cutting plane) for the coding pattern M_3_ under the *y* polarization.**


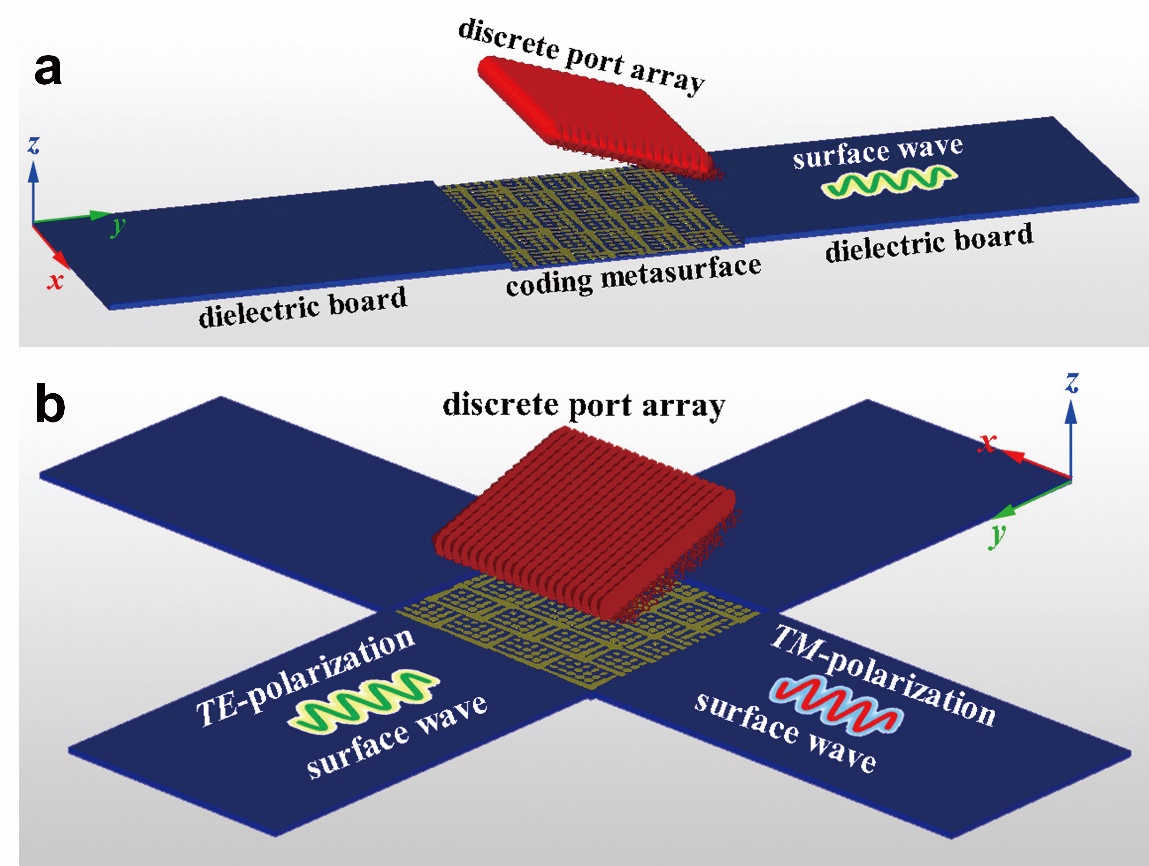


**Supplementary Figure S6 | The simulation models for coding patterns M_4_ and M_5_.** **(a)** For coding pattern M_4_. The discrete port array provides an obliquely incident wave that is tilted 14.5° toward the –*y* direction. **(b)** For coding pattern M_5_. The discrete port array provides an obliquely incident wave that is tilted 14.5° toward the –*y* direction.


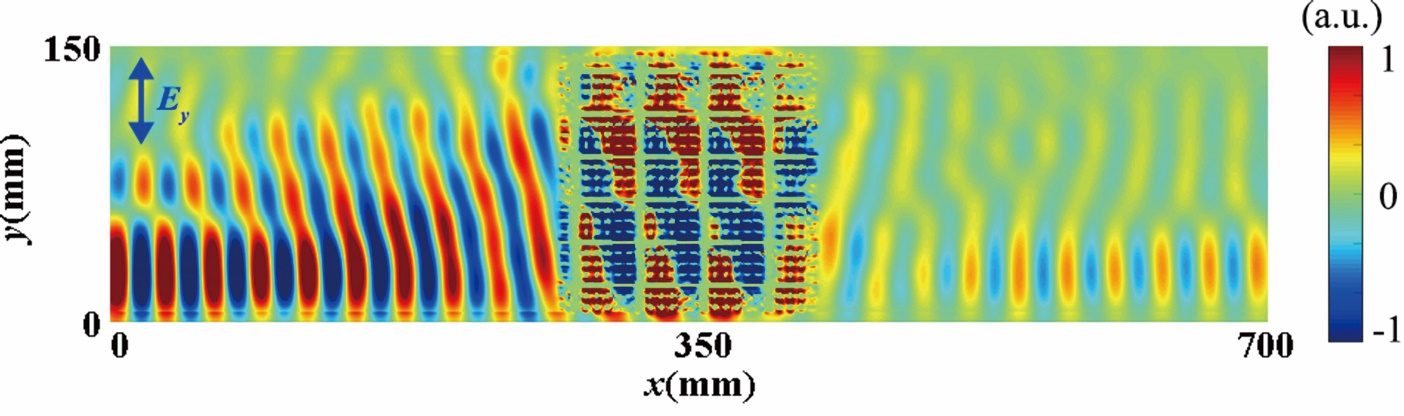


**Supplementary Figure S7 | The electric field distribution for coding pattern M_5_ under the TM polarization. Note that in this case, there is no compensation coding sequence added.**


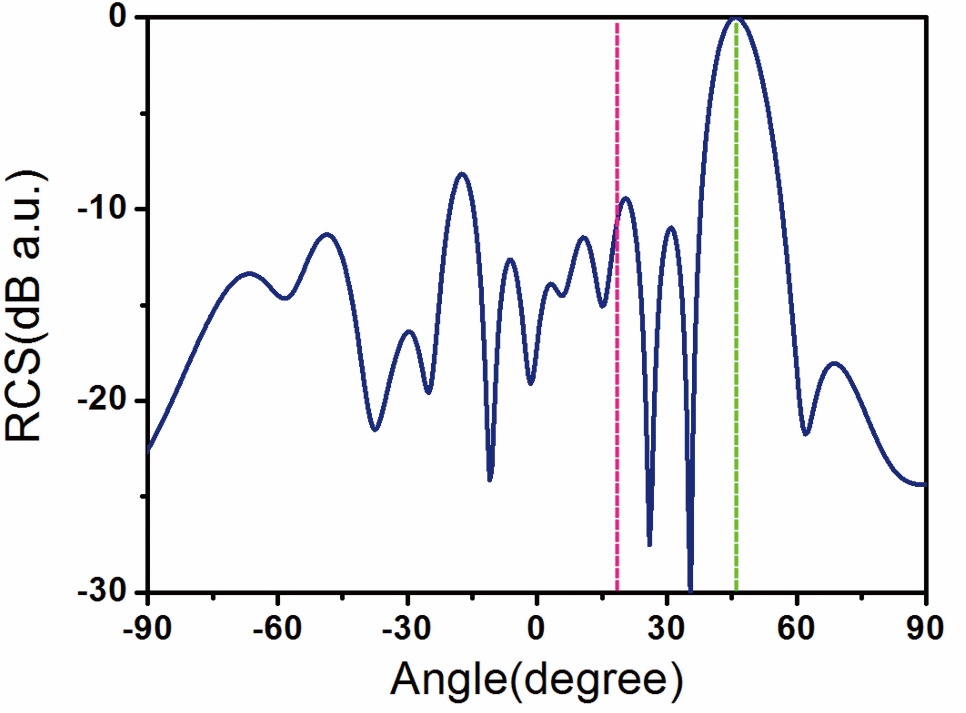


**Supplementary Figure S8 | The simulated far-field radiation pattern for the sample with coding matrix M_1_.**


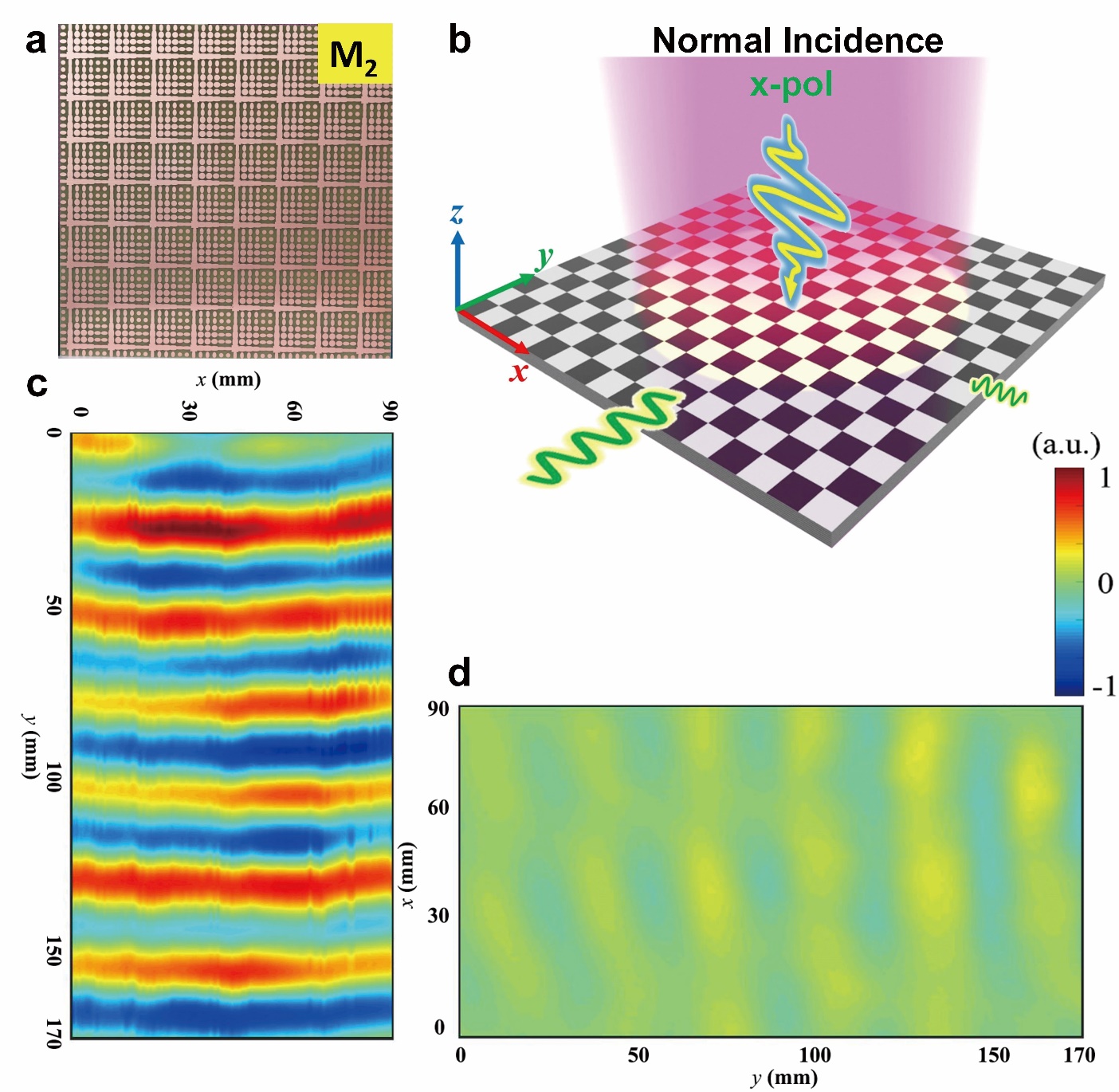


**Supplementary Figure S9 | Experimental results for the near-field measurements of the sample with coding matrix M_2_. (a)** The fabricated sample with coding matrix M_2_. **(b)** Schematic illustration of the experimental characterization of sample M_2_ under the normal incidence. **(c, d)** The electric-field distributions (*E_x_* components) measured at 10 GHz on the dielectric substrate under the normal illuminances with the *x* and *y*-polarizations for coding matrix M_2_, respectively. Here, the scanning area is 90×170mm^2^(*x×y*), and the amplitude intensities in both plots have been normalized.


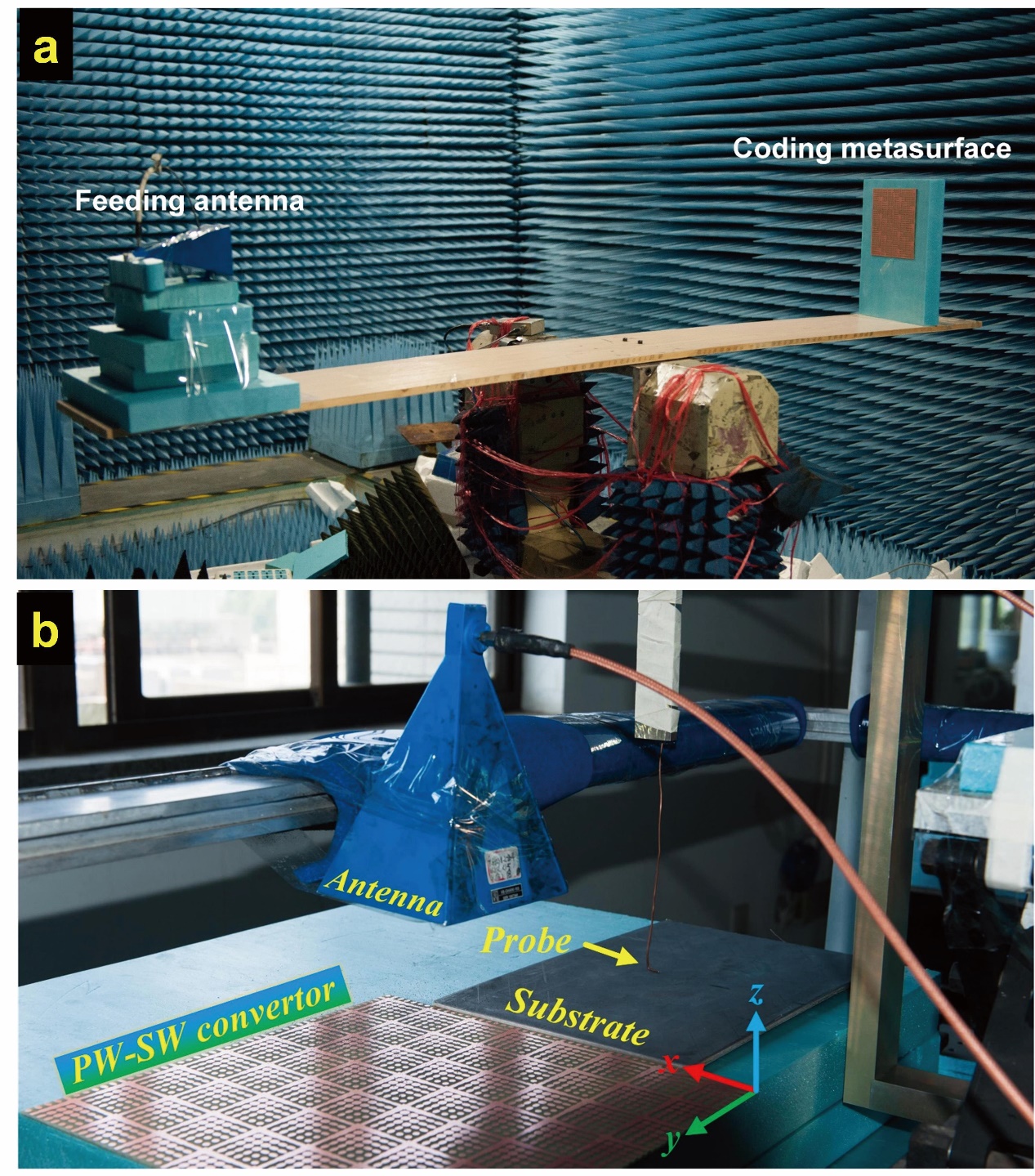


**Supplementary Figure S10 | Experimental setups for far-field radiation pattern and near-field mapping measurements. (a)** The experimental configuration for the far-field radiation pattern measurement in an anechoic chamber. **(b)** The experimental setup for the near-field scanning measurement, where a microwave probe automatically maps the electric field at an altitude of 1 mm above the substrate.
